# Population carrier frequency of hMSH2 and hMLH1 mutations

**DOI:** 10.1054/bjoc.2000.1520

**Published:** 2000-12

**Authors:** M G Dunlop, S M Farrington, I Nicholl, L Aaltonen, G Petersen, M Porteous, A Carothers

**Affiliations:** Colon Cancer Genetics Group, Division of Molecular and Clinical Medicine, MRC Human Genetics Unit, University of Edinburgh, Western General Hospital, Edinburgh, EH4 2XU, UK; Department of Medical Genetics, University of Helsinki, PO Box 21 (Haartmaninkatu 3), Helsinki, FIN-00014, Finland; Departments of Epidemiology and Oncology, Johns Hopkins University, Baltimore, MD, 21205, USA; Department of Clinical Genetics, Western General Hospital, Edinburgh, UK

**Keywords:** colorectal cancer, susceptibility, hMSH2, hMLH1, DNA mismatch repair

## Abstract

Knowledge of population carrier frequency for DNA mismatch repair (MMR) gene mutations would contribute to understanding the burden of cancer due to genetic susceptibility, but robust prevalence estimates are lacking. To estimate carrier frequency, we genotyped a cohort of relatives of mutation carriers and determined their colorectal cancer prevalence. Systematic Finnish and US data were combined with Scottish genotype and cancer prevalence data in a Bayesian calculation. The estimated carrier prevalence in the population aged 15–74 years is 1:3139 (95% Cl = 1:1247–1:7626) and these carriers are at high risk of colorectal and other cancers. © 2000 Cancer Research Campaign http://www.bjcancer.com


*British Journal of Cancer* (2000) **83**(12), 1643–1645
© 2000 Cancer Research Campaign
doi: 10.1054/ bjoc.2000.1520, available online at http://www.idealibrary.com on IDEAL®

Short Communication

### Population carrier frequency of hMSH2 and hMLH1 mutations

**MG Dunlop[1], SM Farrington[1], I Nicholl[1], L Aaltonen[2], G Petersen[3], M Porteous[4] and A Carothers[1]**

[1]Colon Cancer Genetics Group, MRC Human Genetics Unit, University of Edinburgh, Division of Molecular and Clinical Medicine, Western General Hospital, Edinburgh EH4 2XU, UK; [2]Department of Medical Genetics, University of Helsinki, PO Box 21 (Haartmaninkatu 3), FIN-00014 Helsinki, Finland; [3]Departments of Epidemiology and Oncology, Johns Hopkins University, Baltimore, MD 21205, USA; [4]Department of Clinical Genetics, Western General Hospital, Edinburgh, UK

**Summary** Knowledge of population carrier frequency for DNA mismatch repair (MMR) gene mutations would contribute to understanding the burden of cancer due to genetic susceptibility, but robust prevalence estimates are lacking. To estimate carrier frequency, we genotyped a cohort of relatives of mutation carriers and determined their colorectal cancer prevalence. Systematic Finnish and US data were combined with Scottish genotype and cancer prevalence data in a Bayesian calculation. The estimated carrier prevalence in the population aged 15–74 years is 1:3139 (95% CI = 1:1247–1:7626) and these carriers are at high risk of colorectal and other cancers. © 2000 Cancer Research Campaign http://www.bjcancer.com

**Keywords**: colorectal cancer; susceptibility; hMSH2; hMLH1; DNA mismatch repair

People with mutations in DNA mismatch repair genes have a substantial risk of colorectal and other cancers (Vasen et al, 1996; Dunlop et al, 1997; Aarnio et al, 1999; Lynch and de La Chappelle, 1999). However, robust estimates of population carrier frequency are not available because case ascertainment in most reported studies has been biased towards probands with an established family history. Although mutation carriers may have family histories amounting to hereditary nonpolyposis colorectal cancer (HNPCC), a strong family history of colorectal cancer may be lacking in a substantial proportion of people with proven mutations (Farrington et al, 1998; Wijnen et al, 1998). Hence, population prevalence of mismatch repair gene mutations cannot be calculated from studies employing family history ascertainment.

Knowledge of carrier frequency in the population is of considerable importance because it informs the application of mutation-testing strategies as well as defining the contribution of defective DNA mismatch repair to overall colorectal cancer incidence. Here, we determined genotype for hMLH1 and hMSH2 for a cohort of relatives where bias due to family history ascertainment has been minimized. We incorporated these data and the colorectal cancer prevalence for mutation carriers in a Bayesian calculation, along with age-at-onset and mutation data from two systematic series of colorectal cancer patients from Finland and the USA, in order to estimate population carrier frequency of hMLH1 and hMSH2 mutations.

### METHODS

We determined the hMLH1 and hMSH2 genotype of all surviving relatives of Scottish probands with early-onset colorectal cancer, whose mutations were previously reported by us (Dunlop et al, 1997). These six probands with documented mutations were ascertained on the basis of being affected by colorectal cancer when aged less than 30 years at diagnosis, irrespective of family history. Mutation screening was also directed by assessment of tumour microsatellite instability (Dunlop et al, 1997). Thus, relatives of these probands comprise a cohort where colorectal cancer prevalence is not biased by pre-selection on the basis of a cancer family history. We defined the pedigrees fully by face-to-face interviews and a genealogical search of Scottish central records. Colorectal cancer prevalence was determined for mutation carriers within the families by combining information obtained at interview and cancer data from Scottish central records.

Genotypes for family members were determined from DNA or RNA purified from peripheral blood leukocytes. hMSH2 and hMLH1 genotypes were determined by DNA sequencing of PCR products amplified from genomic templates, by specifically designed PCR/restriction digestion assays or by cDNA sequencing, as appropriate for each respective mutation. Sequencing was done using an ABI 377 automated sequencer. Sequencing primers are lodged at: http://www.hgu.mrc.ac.uk/Users/Malcolm.Dunlop/MMRprim. htm.

To provide estimates of population carrier rates in the age group 15–74 years, we used Bayes' rule to derive the following expression for population carrier frequency:

[(carrier frequency in colorectal cancer patients)
× (population prevalence of colorectal cancer)]/
(prevalence of colorectal cancer in carriers)

Each component of the equation is independent and requires data that is unbiased by family history status. We restricted analysis to people aged 15–74 years, in view of surveillance issues and because population cancer prevalence data outwith this age range are less robust.

*Received 13 December 1999*
*Revised 11 August 2000*
*Accepted 6 September 2000*

*Correspondence to:* M Dunlop

Colorectal cancer prevalence for genotyped family members, together with Scottish National Cancer Registry data were incorporated into the expression. Carrier frequency in colorectal cancer patients was determined by combining hMSH2/hMLH1 mutation prevalence data and age distribution from systematically collected cohorts from Finland (Aaltonen et al, 1998) and Baltimore (Liu et al, 1995). Only patients within the 15–74 year age groups from these cohorts were included, to accord with the age selection of the Scottish relatives and population groups. This age restriction is essential for valid comparison within the Bayesian calculation. A Medline search did not identify any other systematic studies of hMSH2 and hMLH1 mutation prevalence in unselected cancer cohorts.

The study was subject to local ethical approval by Lothian and Borders Ethics Committee and to approval by the Multi-Centre Research Ethics Committee for Scotland.

### RESULTS

There were 110 surviving family members aged 15–74 years in the families of five of the probands originally reported by us (Dunlop et al, 1997). Pedigree analysis for the sixth proband was impossible due to non-paternity. Probands themselves were excluded, since they were selected on the basis of having previously developed cancer. Furthermore, surviving parents or grandparents were also excluded from the analysis to avoid potential biases of age and reproductive fitness. There were 48 (44%) relatives aged 15–74 years (mean age = 37.7, SD = 13.0) who carried the same mutation as the respective proband and 62 relatives (mean age = 42.1, SD = 15.0) carried only wild type alleles. Seven mutation carriers (14.6%) developed colorectal cancer within the 10-year period ending 1995 (mean age = 46.1, SD = 11.0). Colorectal cancer prevalence was calculated from 1995 Scottish population data when there were 3838547 people aged 15–74 years (mean age = 42.2, SD = 16.3) (Registrar General for Scotland, 1998). In all, 6712 (0.17%) of the Scottish population aged 15–74 years, who were alive in 1995 had developed colorectal cancer within the previous 10 years (mean age = 64.0, SD = 8.9) (Harris et al, 1998). There were 414 patients aged 15–74 years in the combined Finnish/Baltimore cohort and 11 (2.66%) had germline hMSH2 or hMLH1 mutations (Table 1).

By applying these values as parameters in the formula that we devised, we estimate that the population carrier frequency is 319 per million (95% CI = 131–802 per million assuming that the logarithm of the estimate is normally distributed) or 1:3139 of the population aged 15–74 years. The estimate appears robust because the age distributions of the Scottish, Finnish and Baltimore cohorts are very similar, as are the Scottish population and relatives from the Scottish families whom we established were non-carriers.

**Table 1** Mutation prevalence and age distribution of cohorts from Finland and Baltimore used in the analysis

| | CRC patients aged 15–74 years | | | | |
|---|---|---|---|---|---|
| | Number | Mean age (years) | SD | Carrier | % |
| Aaltonen et al | 337 | 62.1 | 9.7 | 10 | 3.0 |
| Liu et al (Caucasians) | 77 | 60.2 | 11.3 | 1 | 1.3 |
| Combined | 414 | 61.7 | 10.1 | 11 | 2.7 |

### DISCUSSION

This study provides an estimate of carrier frequency of mutations in hMLH1 and hMSH2 based on systematically collected data that is not subject to bias due to family history ascertainment. By applying the estimated carrier frequency to demographic data from the UK (Registrar General for Scotland, 1999; UK Government Statistical Service, 1999), Finland (Population Statistics Finland, 1999) and the USA (US Bureau of Census, 1999) we estimate that, pro rata, there are 13925 people, 1228 people and 62845 people alive in the age group 15–74 years who carry mutations of hMSH2 or hMLH1 in these three populations respectively. It should be noted that there will be additional mutation carriers within these populations who are aged less than age 15 years, and a small number of people aged 75 years or older, but data available to us would not afford reliable estimates in these age groups. Since the risk of colorectal and other cancers is very high for gene carriers (Dunlop et al, 1997), there is substantial rationale in strategies aimed at presymptomatic carrier detection, so that preventive measures might be instigated.

This analysis may underestimate carrier frequency for a number of reasons. The estimate incorporates data over a time period when relatives were not offered surveillance and so a proportion succumbed early to cancer, prior to completing their family. Surveillance and early detection will reduce this reproductive disadvantage to carriers and consequently increase carrier frequency. Underestimation of true carrier rate may also arise as a consequence of the mutation detection strategy used in the Scottish, Finnish and Baltimore studies (Liu et al, 1995; Aaltonen et al, 1998). In each of these cohorts, tumour microsatellite instability was used to select patients for germline mutation searching. However, we have previously shown that 14% of hMSH2/hMLH1 carriers (Farrington et al, 1998) have tumours that do not exhibit microsatellite instability. Furthermore, germline mutations in another MMR gene, hMSH6, are frequently associated with tumours that do not exhibit microsatellite instability (Wijnen et al, 1999; Wu et al, 1999). Hence, although useful for targeting resources, restricting mutation analysis of MMR genes to patients who have microsatellite-unstable tumours may underestimate mutation prevalence in any given cancer cohort. Finally, the families that are the subject of this analysis were ascertained from probands who developed colorectal cancer when aged < 30 years (Dunlop et al, 1997). It is possible that such an extreme phenotype might be a source of bias towards highly penetrant mutations. Lower penetrance mutations are likely to be more prevalent. Since incorporating all of these confounding factors tends to increase the estimated carrier frequency, we are confident that 1:3139 is a minimal estimate for the population in the age range 15–74 years.

We assumed that Finnish and Baltimore data could be applied to the Scottish population. This seems reasonable since the age distribution and colorectal cancer incidence in the three populations is similar. Although every population is, to a certain extent, unique, distortion of the estimate due to differences in the populations considered here are likely to be trivial. Furthermore, we found that the age distributions of the Scottish, Finnish and Baltimore cancer cohorts were also very similar, again supporting the validity of incorporating data from the three population groups in the Bayesian calculation.

The precision of the estimate will improve with ongoing studies of unselected families. Notwithstanding these qualifications, the

*© 2000 Cancer Research Campaign*

estimated population carrier frequency presented here suggests that carrier detection programmes should be incorporated into strategies aimed at reducing CRC mortality, particularly since mutation screening costs are decreasing with technological advances. The lifetime risk of a variety of malignancies for carriers is around 90% and the risk of colorectal and endometrial cancer is particularly well documented (Vasen et al, 1996; Dunlop et al, 1997; Aarnio et al, 1999). Hence, targeting surveillance to those who most need it could make an appreciable impact on reducing overall cancer mortality.

It is important to note that this estimate of carrier frequency is restricted to hMLH1 and hMSH2 mutations. Recent data indicate that mutations of hMSH6 account for an appreciable proportion of HNPCC-like families (Wijnen et al, 1999) and so there seems to be a strong case for devising population-based strategies for detection of germline carriers of mutations in these three mismatch repair genes, in view of the potential for cancer prevention.

### ACKNOWLEDGEMENTS

The work was supported by the following grants in Scotland, Cancer Research Campaign (SP2326/0101, SP2326/0201), Scottish Health Department (K/MRS/50/C2417), Scottish Hospitals Endowment Research Trust (SHERT 1331) and Tenovus Scotland. MGD was funded by a Medical Research Council Clinician Scientist Fellowship and SMF by a Royal Society of Edinburgh Personal Research Fellowship. In Finland, grant funding was by European Commission contract BMH4-CT96-0772 and, in the USA by National Institute of Health grant R01 CA63721. We acknowledge the substantial contributions to this work by Bert Vogelstein, Albert de la Chapelle and their respective laboratories. They contributed to the work both directly as well as by collaboration and provided support and advice. We are indebted to Alison Fordyce of the Medical Research Council Registry for tireless work in pedigree-tracing in Scottish Central Records and to Karen Campbell, Nicola Bradshaw and Pauline Pearson for counselling and sample collection. We also acknowledge the key role of the Information and Statistics Division of the Scottish Health Service in providing population-based data in Scotland and in particular we acknowledge Helen Brown and Roger Black.

### REFERENCES

Aaltonen LA, Salovaara R, Kristo P, Canzian F, Hemminki A, Peltomaki P, Chadwick RB, Kaariainen H, Eskelinen M, Jarvinen H, Mecklin JP and de la Chapelle A (1998) Incidence of hereditary nonpolyposis colorectal cancer and the feasibility of molecular screening for the disease. *N Engl J Med* **338**: 1481–1487

Aarnio M, Sankila R, Pukkala E, Salovaara R, Aaltonen LA, de la Chapelle A, Peltomaki P, Mecklin JP and Jarvinen HJ (1999) Cancer risk in mutation carriers of DNA-mismatch-repair genes. *Int J Cancer* **81**: 214–218

Dunlop MG, Farrington SM, Carothers AD, Wyllie AH, Sharp, Burn J, Liu B, Kinzler KW and Vogelstein B (1997) Cancer risk associated with germline DNA mismatch repair gene mutations. *Hum Mol Genet* **6**: 105–110

Farrington SM, Lin-Goerke J, Wang Y, Burczak J, Robbins DJ and Dunlop MG (1998) Systematic analysis of DNA mismatch repair genes in colon cancer patients and controls. *Am J Hum Genet* **63**: 749–759

Harris V, Sandbridge AL, Black RJ, Brewster DH, and Gould A (1998) Cancer Registration Statistics Scotland 1986–1995. Edinburgh, ISD Scotland Publications: Edinburgh http://www.show.scot.nhs.uk/publications/isd/cancer-registration

Liu B, Farrington SM, Petersen GM, Hamilton SR, Parsons R, Papadopoulos, Fujiwara T, Jen J, Kinzler KW, Wyllie AH, Vogelstein B and Dunlop MG (1995) Genetic instability occurs in the majority of young patients with colorectal cancer. *Nat Med* **1**: 348–352

Lynch HT and de la Chapelle A (1999) Genetic susceptibility to non-polyposis colorectal cancer. *J Med Genet* **36**: 801–818

Population Statistics Finland (1999) http://www.stat.fi/tk/tilsivue.html

Registrar General for Scotland (1998) Annual Report of the Registrar General http://www.open.gov.uk/gros/98sect2.htm

US Bureau of Census data (1999) http://www.census.gov/population/projections/nation/nas/npas9600.txt

UK Government Statistical Service (1999) Official Statistics for the United Kingdom http://www.statistics.gov.uk/stats/ ukinfigs/ukinfig.htm

Vasen HF, Wijnen JT, Menko FH, Kleibeuker JH, Taal BG, Griffioen G, Nagengast FM, Meijers-Heijboer EH, Bertario L, Varesco L, Bisgaard ML, Mohr J, Fodde R and Khan PM (1996) Cancer risk in families with hereditary nonpolyposis colorectal cancer diagnosed by mutation analysis. *Gastroenterology* **110**: 1020–1027

Wijnen JT, Vasen HF, Khan PM, Zwinderman AH, van der Klift, Mulder A, Tops C, Moller P and Fodde R (1998) Clinical findings with implications for genetic testing in families with clustering of colorectal cancer. *N Engl J Med* **339**: 511–518

Wijnen J, de LW, Vasen H, van dK, Moller P, Stormorken A, Meijers-Heijboer H, Lindhout D, Menko F, Vossen S, Moslein G, Tops C, Brocker-Vriends A, Wu Y, Hofstra R, Sijmons R, Cornelisse C, Morreau H and Fodde R (1999) Familial endometrial cancer in female carriers of MSH6 germline mutations. *Nat Genet* **23**: 142–144

Wu Y, Berends MJW, Mensink RGJ, Kempinga C, Sijmons RH, van der Zee AGJ, Hollema H, Kleibeuker JH, Buys CHCM and Hofstra RMW (1999) Association of hereditary nonpolyposis colorectal cancer related tumors displaying low microsatellite instability with MSH6 germline mutations. *Am J Human Genet* **65**: 1291–1298

© 2000 Cancer Research Campaign

